# Post-operative rehabilitation and nutrition in osteoarthritis

**DOI:** 10.12688/f1000research.4178.3

**Published:** 2016-01-06

**Authors:** Giuseppe Musumeci, Ali Mobasheri, Francesca Maria Trovato, Marta Anna Szychlinska, Rosa Imbesi, Paola Castrogiovanni

**Affiliations:** 1 Department of Biomedical and Biotechnological Sciences, Human Anatomy and Histology Section, School of Medicine, University of Catania, Catania, 95123, Italy; 2 Faculty of Health and Medical Sciences, University of Surrey, Guildford, Surrey, GU2 7XH, UK; 3 Arthritis Research UK Centre for Sport, Exercise and Osteoarthritis, Nottingham University Hospitals, Nottingham, NG7 2UH, UK; 4 Center of Excellence in Genomic Medicine Research (CEGMR), King Fahd Medical Research Center (KFMRC), King AbdulAziz University, Jeddah, 21589, Saudi Arabia; 5 Department of Clinical and Experimental Medicine, Internal Medicine Division, School of Medicine, University of Catania, Catania, 95123, Italy

**Keywords:** Osteoarthritis, Rehabilitation, Exercise

## Abstract

Osteoarthritis (OA) is a degenerative process involving the progressive loss of articular cartilage, synovial inflammation and structural changes in subchondral bone that lead to loss of synovial joint structural features and functionality of articular cartilage. OA represents one of the most common causes of physical disability in the world. Different OA treatments are usually considered in relation to the stage of the disease. In the early stages, it is possible to recommend physical activity programs that can maintain joint health and keep the patient mobile, as recommended by OA Research Society International (OARSI) and European League Against Rheumatism (EULAR). In the most severe and advanced cases of OA, surgical intervention is necessary. After, in early postoperative stages, it is essential to include a rehabilitation exercise program in order to restore the full function of the involved joint. Physical therapy is crucial for the success of any surgical procedure and can promote recovery of muscle strength, range of motion, coordinated walking, proprioception and mitigate joint pain. Furthermore, after discharge from the hospital, patients should continue the rehabilitation exercise program at home associated to an appropriate diet. In this review, we analyze manuscripts from the most recent literature and provide a balanced and comprehensive overview of the latest developments on the effect of physical exercise on postoperative rehabilitation in OA. The literature search was conducted using PubMed, Scopus, Web of Science and Google Scholar, using the keywords ‘osteoarthritis’, ‘rehabilitation’, ‘exercise’ and ‘nutrition’. The available data suggest that physical exercise is an effective, economical and accessible to everyone practice, and it is one of the most important components of postoperative rehabilitation for OA.

## Introduction

Osteoarthritis (OA) is a degenerative disease of load-bearing synovial joints ^1– 5^. Knee OA is the most common type of OA ^6^ and represents one of the most common causes of physical disability in the world ^7– 9^. Deterioration of the articular cartilage is the main problem associated with OA with consequent chronic pain and functional restriction ^10^. OA can be caused by previous traumas (fractures, ligament tears and meniscal injury), wrong kinematics, obesity, genetics and age, which lead to alterations in the joint cartilage ^2, 8, 10^. Traumatic injury to synovial joints is increasingly considered an important risk factor for the development of post-traumatic OA (PTOA). Traumatic injuries sustained during the lifetime of an individual, combined with normal age-related wear and tear, may conspire to facilitate the progression of degenerative joint diseases and may lead to chronic disability. OA is an insidious disease that typically develops gradually over the years with several symptoms including pain, stiffness, limited range of motion (ROM) in the joint and localized swelling. OA pain usually worsens after physical activity ^3, 11– 13^, while stiffness arises after sitting for prolonged periods of time. As OA progresses, symptoms generally become more severe and then pain can become continuous. Generally, OA occurs when the dynamic steady-state between destructive forces and repair mechanisms alters the joint homeostasis ^7, 14^. For example, the tibiofemoral mechanics and loading patterns, during walking, influence the regional development of the articular cartilage ^8^. Alterations in normal gait mechanics due to trauma, acute injury, ligamentous laxity, weight gain and improper footwear can shift the loading patterns to areas of the articular cartilage not well adapted to accept improper loads ^8^. If patients do not improve with non-invasive therapies or have excessive pain and loss of mechanical function, OA treatment consists of surgical intervention ^15, 16^ and subsequent rehabilitation ^2, 17– 19^. All patients with hip and knee OA should be informed about the objectives of the treatment and educated to the importance of all the measures that unload the damaged joint (lifestyle changes, regular exercise, weight reduction and other). The initial focus should be on self-help and patient-driven treatments rather than on passive therapies delivered by health professionals. Subsequently, emphasis should be placed on encouraging adherence to the regimen of non-pharmacological therapy ^20^, as widely promoted during the last years ( Table 1). Recently, the European League Against Rheumatism (EULAR) proposed 11 evidence-based recommendations for the non-pharmacological management of people with hip or knee OA ^21^. Moreover, the OA Research Society International (OARSI) proposed other evidence-based recommendations, providing guidance to patients and practitioners on the treatments applicable to all individuals with knee OA, as well as therapies, lifestyle diet and exercise interventions that can be considered according to specific patient needs and preferences ^22, 23^. In the present review, we analyze the effects of postoperative rehabilitation exercise program in OA patients treated with surgical procedures. The aim of this review was to underline the importance of exercise combined with an appropriate daily diet in postoperative rehabilitation for OA patients, and to present exercise as an effective and economical accessible to everyone.

**Table 1.** Physical activity recommendations for patients suffering from osteoarthritis.

Type of activity	Examples	Recommendations
LOW-IMPACT AEROBICS	• Brisk walking • Dancing • Cycling • Group exercise • Swimming Gardening • Water aerobics	2 hours and 30 minutes moderate- intensity or 1 hour and 15 minutes high-intensity.
MUSCLE STRENGTHENING	• Working with resistance bands • Weight training • Calisthenics	2 or more days per week.
BALANCE	• Standing on one foot • Tai chi	3 days per week.

## Materials and methods

In this narrative review, we analysed articles from the most recent literature, providing a balanced and comprehensive overview of the most important discoveries on pathogenesis and therapeutic approaches for osteoarthritis in to the context of post-operative rehabilitation and nutrition. Subsequently, the selected articles were divided in “Morphological aspects of osteoarthritis”, “First step postoperative rehabilitation in OA”, “Second step postoperative rehabilitation in OA” and “Nutrition in postoperative rehabilitation in OA”, to provide interested researchers with a detailed and schematic overview of all the recent studies on osteoarthritis. Key words included osteoarthritis, knee OA, hip OA, hand OA, physical therapy, physiotherapy, rehabilitation, exercise, nutrition, post-operative rehabilitation in OA, physical activity, nutrition in postoperative rehabilitation in OA. The searches were limited to studies published in English that included human and animal studies related to OA, rehabilitation and nutrition. Study designs included narrative, systematic and meta-analyses reviews, original articles and randomized controlled trials (RCTs). We excluded protocols, abstracts without a full article, conference proceedings, papers that replicated data from another article, and studies of outcomes after surgery (such as rehabilitation following joint replacement), oral or injectable medications, neutraceuticals and dietary weight loss (unless accompanied by exercise). We started the literature search in March 2014 to December 2015 on PubMed, Scopus, Web of Science and Google Scholar. The initial searches revealed a total of 190 articles with 77 (from 1995 to 2015) of these deemed to meet the eligibility criteria, considered appropriate for the purpose of the review. These included 16 narrative, systematic and meta-analyses reviews, 3 RCTs and 58 original articles. The other papers, have not been considered as they resulted outside the scope of the research.

## Morphological aspects of osteoarthritis

Cartilage is the most commonly studied tissue in the joint in the context of OA research. It is a unique load-bearing connective tissue with viscoelastic and compressive properties that are largely due to the presence of extracellular matrix, mainly composed of collagen type II and the proteoglycan aggrecan ^9, 17, 18, 24^. OA is a degenerative process involving the progressive loss of structural features and functionality of the articular cartilage caused by an imbalance between anabolic and catabolic processes in the cartilage tissue, so that cartilage degradation exceeds reparative processes and OA progresses ^10, 14, 25^. Moreover, the articular cartilage and the subchondral bone are two mechanically and biologically intertwined tissues, which suffer changes during the OA process ^26^. The vascular system of subchondral bone provides the articular cartilage with nutrition but in adulthood the articular cartilage is no longer able to obtain nutrition from the bony vascular supply and this could impact its ability to recover from injury ^27^. Furthermore, there are clear evidences of an association between subchondral bone mineral density and osteoarthritis ^28^. Generally, the surface of healthy hyaline cartilage appears white, shiny, elastic and firm. In contrast, OA cartilage shows a dull and irregular surface with discoloration, softening, and often with increased production of synovial fluid ^29^. In advanced OA the cartilage shows signs of rupture; the cartilage surface is rough and broken by fissures and cracks which can reach down to the calcified zone ^30^, and chondrocytes are arranged in clusters (especially around fissures) or disappear. The organization of cartilage is widely disordered and replaced by fibro-cartilaginous, scar-like tissue with fibroblast-like cells ^31^. As described in detail by several authors, the development of a rheumatoid-like ‘pannus’ of various extents can overlay the damaged cartilage tissue ^32, 33^. The extent of damage to the articular cartilage depends on the joint surface area, which is exposed to different loading patterns and conditions in distinct regions ^29^.

## First step post-operative rehabilitation in OA

Postoperative rehabilitation is crucial for the success of any surgical procedure ^34^. It has the purpose of recovering muscle strength, range of motion, coordination in walking and mitigation of the pain. The postoperative rehabilitation program usually starts 48 hours after the surgery procedure as a result of the clinical evaluation of each specific case of OA. The rehabilitation is often long because of the time necessary for the cartilage cells to adapt and mature into repair tissue. Cartilage is a slow adapting tissue, indeed it undergoes 75% adaptation in approximately 2 years ^35^. When the rehabilitation period is too short, the cartilage repair might be put under too much stress, causing the repair to fail ^34^. The type of postoperative exercise program depends on the injury. Experimental and clinical studies demonstrate that early, controlled mobilization is superior when compared to immobilization for primary treatment of acute musculoskeletal soft-tissue injuries and postoperative management. Early mobilization helps return the patients more quickly to physical activity, reduces persistent swelling, restores stability, restores ROM, and improves patient satisfaction with the rehabilitation outcome ^36^.

A postoperative rehabilitation exercise program should be personalized and based on the type of surgical procedure, location, size and depth of the lesion, in order to facilitate the healing process ^37^, as well as on the age and medical condition.

Arthroscopic procedures, such as chondroplasty (a procedure based on the use of a graft of cartilage tissue) or microfracture (a technique using to perform microfractures into the intracortical bone so as to involve the neighboring mesenchymal stem cells in order to form a combination of cartilage and fibrous tissue with varying amounts of type-II collagen content), may resolve faster than osteochondral autograph transplantation (OATS) (a technique that involve transplantation of small cylindrical osteochondral grafts harvested from the articular surface and transferred to create a resurfaced area in the lesion) or autologous chondrocyte implantation (ACI) (a procedure that has the aim of repairing chondral defects by implanting cartilage cells) that involve larger incisions, requiring a slower exercise rehabilitation program ^37, 38^. Since immobilization and unloading result in proteoglycan loss in articular cartilage and gradual weakening, controlled weight bearing and ROM are essential to facilitate the healing process and to prevent degeneration ^25, 39, 40^. Furthermore, controlled compression and decompression forces during weight bearing nourish the articular cartilage and induce molecular signals necessary to produce an optimal extracellular matrix ^39^. A force platform is a useful tool in the rehabilitation program to perform limited weight-bearing activities facilitate a normal gait pattern and enhance strength, proprioception, and balance ^37^. The postoperative rehabilitation exercise program includes performance of motion exercises and muscle strengthening with any ambulatory aids (walker, sticks, forearm crutches), training in postural changes and in the execution of stairs. During rehabilitation, the passive range of motion (PROM) activities, in a limited ROM, are also indicated to nourish the healing articular cartilage and prevent the formation of adhesions ^40^. Continuous passive motion (CPM) enhances cartilage healing and long-term outcomes following articular cartilage procedures ^40^. As the lesion heals and symptoms decrease, the ROM is modified to allow greater muscle strengthening over a greater range of movement ^37^. With surgical procedures, particularly with the OATS and ACI, because of the large incision and extensive soft tissue trauma, arthrofibrosis could take place and rehabilitation can avoid this event ^37^. When the surgical procedure has implanted a prosthesis, depending on the type of prosthesis, the use of special machines for the passive flexion-extension of the joint is advisable. Symptoms, such as pain and effusion, could cause the inhibition of the muscles, so electrical muscle stimulation and biofeedback are complementary with the rehabilitation exercise program to promote the active contraction of musculature ^41^. Stretching exercises should be included as the patient progresses to advanced phases of rehabilitation ^37^. As the patient returns to functional activities, it is important to increase gradually the amount of stress applied to the treated joint, to provide a stimulus for healing to cartilage tissues without causing damage ^37^. The rehabilitation exercise program following surgical procedures for OA is fundamental to the long-term success and functional outcome of patients involved ^37, 42^ ( Table 2).

**Table 2.** First step postoperative rehabilitation in OA. Schematic representation of primary rehabilitation activities that should be included in post-operative rehabilitation program soon after the surgery.

PHASE	TYPE OF ACTIVITY	EFFECTS
**FIRST STEP**	CONTROLLED WEIGHT BEARING	Nourishes the articular cartilage and provides molecular signals necessary to produce an optimal extracellular matrix.
	RANGE OF MOTION (ROM)	Facilitates healing process and prevents degeneration.
	FORCE PLATFORM	Facilitates a normal gait pattern and enhances strength, proprioception, and balance.
	PASSIVE RANGE OF MOTION (PROM)	Nourishes the healing articular cartilage and prevents the formation of adhesions.
	CONTINUOUS PASSIVE MOTION (CPM)	Enhances cartilage healing and long-term outcomes.
	ELECTRICAL MUSCLE STIMULATION AND BIOFEEDBACK	Promotes the active contraction of musculature.
	STRETCHING EXERCISES (in advanced phases of rehabilitation)	Increases the amount of stress applied to the joint and provides a stimulus for cartilage tissue healing.
* In case of **prosthesis**	PASSIVE FLEXION-EXTENSION (with use of special machines)	Facilitates healing process.

## Second step post-operative rehabilitation in OA

Following hospital discharge, the patient should continue the rehabilitation exercise program at home. The physiotherapist will indicate and teach the exercises to be carried out independently, aimed at maintaining a good muscular and articular quality. Patients surgically treated for OA often suffer from pain and have problems during everyday activities, and physical activity could attenuate these deficits ^43^. Strengthening exercises, aerobic exercises or both together, show positive effects for both pain and physical function ^43, 44^. However, data from literature show that the long-term benefits of exercise have no significant effect on pain or physical function after 6 months, except when booster sessions are implemented ^45^.

*Resistance exercise* decreases pain and increases physical function, reducing disability ^46^. It includes loads, repetitions, movement speed and frequency of sessions, and often is supported by the use of machines or free weights ^47^. Strength, ROM, pain throughout the range of motion and the possibility of patient to have access to the necessary equipment for exercise should be considered for a resistance exercise program ^47^. When access to machines is too expensive for the patient, an exercise program should still be performed at home ^48– 50^. The resistance exercise program should be performed 3 days per week, with 2–3 sets per exercise at 8–15 repetitions per set ^47^, and loads should vary from high to low ^50^. The patient’s tolerance should take into account the initial resistance loads and the joint ROM ^47^. The resistance loads or number of sessions per week should increase as the patient acquires strength and confidence ^47^. Resistance exercise increases muscle strength ^48, 49^ and in a period of 2–9 months of progressive exercise, pain could decrease by 42–43% ^48, 49^. Isokinetic torque can increase further after greater resistance exercise intensity ^48^. These data support the idea that improvements in symptoms and function are directly related to exercise intensity and that higher intensity resistance exercise sustains muscle strength and preserves functionality ^49^.

*Aerobic exercise* includes several activities such as walking, cycling or the use of a seated stepper machine. It has beneficial effects on joint mobility and pain, and it improves the functional status of their general mobility and respiratory capacity ^34, 51^. Although modality and dosage are currently not well defined, aerobic exercise program should take into account age, mobility, co-morbidities and preferences ^34^. The exercise bike is a helpful tool for exercising at home. Aquatic exercise seems not to have effects on walking ability or joint ROM ^52^, so it should be considered as an optional activity for exercise program ^34, 52^. Land-based exercise and aerobic exercise show higher beneficial effects for pain and function compared with aquatic exercise and strengthening exercise ^53^. A combination of both aerobic training and strengthening exercise could be an optimal choice to decrease impairments ^54^. The beneficial effects of exercise programs are mostly related to the adherence and constancy of patients to the program and the number of sessions, while variations in the delivery, content and dosage do not influence the outcome ^43^. Data from literature show that exercise programs have short-term benefits in reducing pain and improving physical function, but they do not persist in the long term without adherence to the program ^43, 45, 51^. Strategies to increase long-term adherence to exercise are necessary to maximize the benefits of exercise program ^43^. Self-efficacy is also associated with higher adherence and better outcomes ^51^. Finally, the exercise program should be combined with education and behavioral strategies to promote a positive lifestyle change and increase physical activities ^34, 55^ ( Table 3).

**Table 3.** Second step postoperative rehabilitation in OA. Schematic representation of rehabilitation activities that the patients, once discharged from hospital, should keep on at home.

PHASE	TYPE OF ACTIVITY	EFFECTS
**SECOND** **STEP**	STRETCHING EXERCISES	Increases gradually the amount of stress applied to the treated joint, provides a stimulus for healing to cartilage tissues without causing damage and has positive effects for both pain and physical function.
	RESISTANCE EXERCISE	Decreases pain, increases physical function and reduces disability.
	AEROBIC EXERCISE	Has beneficial effects on joint mobility and pain, and improves functional status and respiratory capacity.
	NUTRITIONAL EDUCATION	Improves metabolic homeostasis of cartilage tissue and determines protection against chronicity of the disease.

## Nutrition in postoperative rehabilitation in OA

**Figure 1.**
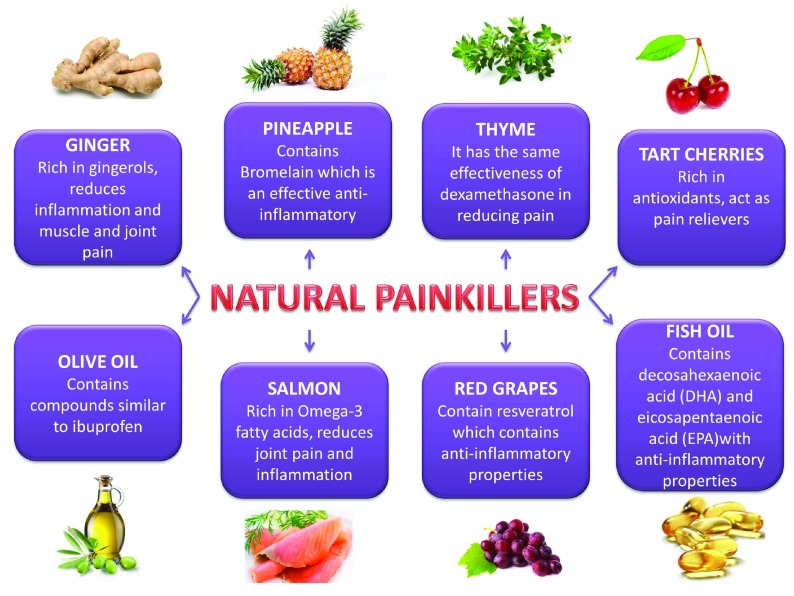
Foods containing compounds with anti-inflammatory and analgesic properties, that may help ease the symptoms of osteoarthritis as well as improve the overall health of patients.

As mentioned above, cartilage is a connective tissue with viscoelastic and compressive properties, largely due to the extracellular matrix, mainly composed of collagen type II and the proteoglycan aggrecan ^9, 17, 18, 24^. In OA, a progressive loss of structural features occurs because of an imbalance between anabolic and catabolic processes in the cartilage tissue ^10, 14, 25^. Therefore, one of the goals of OA postoperative rehabilitation, in addition to the restoration of joint function, is the metabolic homeostasis of cartilage tissue, also obtainable through an appropriate diet. The exercise program in OA postoperative rehabilitation would surely have a greater efficacy if combined with a nutritional education in order to promote a healthier lifestyle. There are numerous foods containing natural anti-inflammatory compounds, which are able to reduce some important symptoms of OA, such as pain. These foods are known as natural painkillers and some of them are illustrated in Figure 1.

The Mediterranean Diet (Med Diet) is the traditional dietary pattern of the Mediterranean areas in the early 1960s ^56^. Olive oil (OO) is the principal source of fat of Med Diet. It is extracted from *Olea europaea* fruits and is rich in monounsaturated fatty acids (MUFAs). The beneficial effects of OO are ascribed to its phytochemicals such as phenolic compounds, tocopherol and carotenoids, that have antimicrobial, antioxidant and anti-inflammatory properties ^57^. Some epidemiological studies reported an association between consumption of diets rich in polyphenols and protection against chronic diseases ^58^, but few studies investigated the effects on cartilage tissue of such compounds that seem to have a potential protective role ^59^. The phenolic compounds present in OO may interact with the inflammatory cascade preventing cellular damage thank to their antioxidant action. In rheumatoid arthritis patients the dietary supplementation with OO improves joint pain and morning stiffness ^60^. Both leaves and fruit of the olive plant are rich in beneficial polyphenols ^61^, among which the most bioactive are oleuropein and hydroxytyrosol ^59^. Oleuropein is a secoiridoid and represents the most important microconstituent of virgin OO for its health implications. It has high antioxidant activity *in vitro*, and its hydrolysis product, oleuropein aglycone, ameliorates resistance to the development of arthritis ^62^. Indeed oleuropein reduces the release of proinflammatory cytokines and leukocytes infiltration in the joints affected by collagen induced arthritis, thus reducing the progression of chronic joint inflammation ^62^. Moreover, when administered after the clinical onset of arthritis, oleuropein reduces swelling and the other clinical manifestations, as well as the histological severity of the disease ^62^. This compound reduced the bone loss and improved inflammation, showing a bone sparing effect, in an animal model of senile osteoporosis ^63^.

Another important phenolic compound is oleocanthal (OLC) that shows anti-inflammatory and neuroprotective properties ^64^. OLC inhibits the cyclooxygenase enzymes in the pathway of prostaglandin biosynthesis in a more potent manner than ibuprofen ^65^. Rutin (quercetin-3-O-rutinoside) is a flavonoid ubiquitously found in plants. Quercetin, the circulating aglycone form of rutin, has the ability to scavenge free radicals ^66, 67^ and the association with oleuropein induces interesting metabolic and structural effects on OA cartilage and synovium, supporting their use in human trials ^59^. The fruits of *Elaeagnus angustifolia* is similar to those of *Olea europaea*, and although belonging to another botanical family, possesses the same anti-inflammatory potential and was showed to be active in female arthritis patients ^68^.

Given its known anti-inflammatory properties, we have recently studied the possible benefits of extra-virgin OO, in association with physical activity on joint disease, in order to evaluate the inflammation and the expression of lubricin in articular cartilage after injury and the consequent occurrence of OA ^16^. In our study, we highlighted that Med Diet and extravirgin OO consumption may help attenuate and resolve inflammation in articular cartilage after injury, preventing OA ^16^.

Moreover, deficiencies of vitamins D ^69, 70^ and K ^71^ increase the risk of development and progression of OA. A recent controlled trial on arthritis patients comparing the exercise and the nutritional interventions, according to the MyPyramid and MyPlate approaches ^72^, showed an improvement also in the nutritional program group, probably due to the weight loss and the increase of motivation to leisure time physical activity ^73^.

In relation to weight loss, a recent study showed that weight loss due to an intensive dietary intervention results in bone loss in overweight and obese, older adults with OA, and that the exercise intervention did not attenuate weight loss-associated reductions in bone mineral density even if the rate of osteoporosis and osteopenia remained unchanged ^74^. Indeed, lowering the fat content typical of the Western diet increases daily physical activity and resting energy expenditure, affecting also the mood, in particular anger and hostility ^75^. Thus, a high consumption of saturated fats might reduce the motivation for physical activity leading to the individual’s propensity for weight gain, which is detrimental especially in patients affected by OA. Therefore, a healthy diet combined to a rehabilitation exercise program could improve the quality of life and the mood of post-surgery patients ^76, 77^.

## Conclusions

The articular joint is a highly complex ‘organ system’ that requires regular maintenance. The immobilization of the joints results in a number of negative physiologic consequences. Severity, mobility, pain, stiffness and radiographic progression may be partly mediated by the level of chronic inflammation in OA patients. In the most severe cases of OA, surgical intervention is necessary. It is essential to combine a postoperative rehabilitation exercise program with surgical interventions in order to restore full function of the involved joint. This is crucial for the success of any articular cartilage surgery procedure, and has the purpose to improve muscle strength, ROM, coordination in walking and mitigate of the pain. After hospital discharge, patients should continue the rehabilitation exercise program at home with strengthening exercises, aerobic exercises or both, combined together with a correct diet, so that positive benefits may be gained in terms of pain control and quality of life. Furthermore, regular physical activity combined with a healthy diet improves physical function, muscular strength and endurance, reduces some OA symptoms and leads to psychological and mood benefits. The goal of the postoperative rehabilitation program in OA is to restore joint function, prevent functional limitations and mitigate the progression of the disease, but it is evident that it would surely have a greater efficacy if combined with a nutritional education in order to promote a healthy lifestyle.
