# Supplementary material for: Post-operative rehabilitation and nutrition in osteoarthritis
Source: F1000Res. 2016 Jan 6;3:116. Originally published 2014 May 23. [Version 3] doi: 10.12688/f1000research.4178.3 (PMC4765713; doi:10.12688/f1000research.4178.3)
Supplement: Peer review history

# Peer review: Referee response for version 3

**DOI:** 10.5256/f1000research.8283.r12177

**Published:** 2016-02-23

**Authors:** Fabio Galbusera

**Affiliations:** 1 Laboratory of Biological Structures Mechanics, IRCCS Galeazzi Orthopedic Institute, Milan, Italy

In the current version, the paper is well written and clear. Despite its narrative nature, the methods used for the literature search are meaningful and well explained. The conclusions are supported by the evidence emerging from the literature review.

I have read this submission. I believe that I have an appropriate level of expertise to confirm that it is of an acceptable scientific standard.

# Peer review: Referee response for version 3

**DOI:** 10.5256/f1000research.8283.r12209

**Published:** 2016-01-29

**Authors:** Ivana Gadjanski

**Affiliations:** 1 R&D Center for Bioengineering - BioIRC, Kragujevac & Metropolitan University Belgrade, Kragujevac, Serbia

After the revision, the manuscript by Musumeci *et al. *is of an acceptable scientific standard.

I have read this submission. I believe that I have an appropriate level of expertise to confirm that it is of an acceptable scientific standard.

# Peer review: Referee response for version 2

**DOI:** 10.5256/f1000research.6514.r11704

**Published:** 2015-12-29

**Authors:** Carolien H. G Bastiaenen

**Affiliations:** 1 Department of Epidemiology, Maastricht University, Maastricht, Netherlands

It is an well-written and informative paper on the topic. The paper underlines the importance of physical expertise on the treatment of OA, combined with an appropriate diet in post-operative situations. Nevertheless I have serious comments on the scientific basis of the review.

The review is not performed in line with a scientific accepted methodology regarding systematic reviews. The search terms only are focused on the topic OA and not on methodological terms. There are no in-and exclusion criteria for the papers formulated. Also, there is no quality assessment of the papers regarding methodological quality or a systematically presented evidence synthesis. Therefore the presentation of the results, discussion and the conclusion is difficult to interpret.

I have read this submission. I believe that I have an appropriate level of expertise to confirm that it is of an acceptable scientific standard, however I have significant reservations, as outlined above.

# Response

**Published:** 2015-12-30

**Authors:** Giuseppe Musumeci

**Affiliations:** University of Catania, Italy

Point by point replies

Reviewer

It is an well-written and informative paper on he topic. The paper underlines the importance of physical expertise on the treatment of OA, combined with an appropriate diet in post-operative situations. Nevertheless I have serious comments on the scientific basis of the review.

Response

The authors thank the reviewer for this comment.

Reviewer

The review is not performed in line with a scientific accepted methodology regarding systematic reviews. The search terms only are focused on the topic OA and not on methodological terms. There are no in-and exclusion criteria for the papers formulated. Also, there is no quality assessment of the papers regarding methodological quality or a systematically presented evidence synthesis. Therefore the presentation of the results, discussion and the conclusion is difficult to interpret.

Response

We thank the reviewer for this comment. Probably, the misunderstanding came from the scope of this paper. Authors intentions were to write a Narrative Review, as now specified in the methodological section, although this type of review has some limitations.

There are two kinds of review articles commonly present in the scientific literature as shown in the following editorial: Rother, ET: Systematic literature review X narrative review. *Acta paul. enferm*. 2007; **20**(2): 5-6. Publisher Full Text.

In summary this editorial says:

Narrative literature review articles are contributions which describe and debate the state of scientific knowledge of a specific field with a critical analysis of the literature published.

These types of review articles do not list the types of databases and methodological approaches used to carry out the review nor the evaluation criteria for inclusion of retrieved articles during databases search, anyway we added in the methodology section more information and details as kindly suggested (inclusion and exclusion criteria and so on) to improve our review and to help readers better understand.

Narrative literature review articles have a key role in continuous learning/training because they provide readers with update knowledge about a specific field of interest, although it does not describe the methodological approach that would permit reproduction of data nor answer to specific quantitative research questions. Results are of a qualitative rather then a quantitative meaning.

Moreover, systemic literature review "is a well planned review to answer specific research questions using a systematic and explicit methodology to identify, select, and critically evaluate results of the studies included in the literature review". Therefore, systematic literature review utilize methodological rigor to prevent the prejudice of a narrative review.

The Authors hope that the reviewer will be satisfied with the changes and revisions made.

Thank you for your time and for considering our paper.

# Peer review: Referee response for version 2

**DOI:** 10.5256/f1000research.6514.r8515

**Published:** 2015-05-07

**Authors:** Ivana Gadjanski

**Affiliations:** 1 R&D Center for Bioengineering - BioIRC, Kragujevac & Metropolitan University Belgrade, Kragujevac, Serbia

The review article by Musumeci *et al*. under the title "Post-operative rehabilitation and nutrition in osteoarthritis" is well written, with appropriate title and abstract. The article largely accomplishes to underline importance of exercise, combined with an appropriate daily diet, in postoperative rehabilitation for osteoarthritis (OA) patients. However, I have several comments which I hope will help the authors to bring across this important message even more efficiently.

Major points:

- It is not very clear how the selection of articles was performed. The authors state: "The literature search was conducted from in March 2014 to April 2015 on PubMed, Scopus, Web of Science and Google Scholar using appropriate keywords (osteoarthritis, rehabilitation, exercise and nutrition). Of approximately 160 papers (original articles, systematic and meta-analysis reviews) only 67 have been chosen and considered appropriate for the purpose of the review. The other papers, have not been considered as they resulted outside the scope of the research." However, out of these 67, only 7 are from 2014 and 3 from 2015. The rest range from 1995-2013 (References). The authors should clarify the selection criteria and perhaps include more of the more recent papers. 
  Suggestions of the papers to include:
  - OARSI Clinical Trials Recommendations: Design and conduct of clinical trials of lifestyle diet and exercise interventions for osteoarthritis
  - Knee osteoarthritis: Clinical connections to articular cartilage structure and function
  - Exercise for osteoarthritis of the knee
  - Regular Exercises in Knee and Hip Osteoarthritis
  - Exercise, nutrition and managing hip fracture in older persons
- The authors state: " *OA is a degenerative process involving the progressive loss of structural features and functionality of the articular cartilage caused by an imbalance between anabolic and catabolic processes in the cartilage tissue, so that cartilage degradation exceeds reparative processes and OA progresses*" - this is not sufficiently precise, since the subchondral bone is also involved in pathogenesis of OA. The authors should also mention this and further on, comment on the effects of weight loss/gain and exercise training on bone mineral density in OA patients.
  Suggested paper: The independent and combined effects of intensive weight loss and exercise training on bone mineral density in overweight and obese older adults with osteoarthritis.

Minor points:

- " *Cartilage is a slow adapting tissue, indeed it undergoes 75% adaptation in approximately 2 years.*" - Reference for this statement? 
- The paragraph under title “ *Second step post-operative rehabilitation in OA*” - the whole paragraph is very repetitive, with the same kind of information provided in the table as well. Can be shortened. 
- It would be good to give short overview of the available surgical procedures to treat OA. The authors mention “ *Arthroscopic procedures, such as chondroplasty or microfracture, may resolve faster than osteochondral autograph transplantation (OATS) or autologous chondrocyte implantation (ACI) that involve larger incisions, requiring a slower exercise rehabilitation program*”, but it would be better if they gave a short description of each procedure (not more than a sentence long).

I have read this submission. I believe that I have an appropriate level of expertise to confirm that it is of an acceptable scientific standard, however I have significant reservations, as outlined above.

# Response

**Published:** 2015-06-08

**Authors:** Giuseppe Musumeci

**Affiliations:** University of Catania, Italy

Thank you for your review and for the comments. Comments from reviewers are all valuable and very helpful for revising and improving our paper. We have studied the comments carefully and have made corrections which we hope meet with your approval.

Point by point replies

- **Reviewer: **It is not very clear how the selection of articles was performed. The authors state: "The literature search was conducted from in March 2014 to April 2015 on PubMed, Scopus, Web of Science and Google Scholar using appropriate keywords (osteoarthritis, rehabilitation, exercise and nutrition). Of approximately 160 papers (original articles, systematic and meta-analysis reviews) only 67 have been chosen and considered appropriate for the purpose of the review. The other papers, have not been considered as they resulted outside the scope of the research." However, out of these 67, only 7 are from 2014 and 3 from 2015. The rest range from 1995-2013 (References). The authors should clarify the selection criteria and perhaps include more of the more recent papers.
**Response: **The authors thank the reviewer for this comment. Perhaps we have not been clear, we meant to say that we have made the literature search in the period March 2014 - April 2015, but the period considered in the selection of the references is much larger (1995 – 2015). Thank you for letting us know about the possible misinterpretation. We have corrected the text to be clearer. Thank you for the papers suggested, we included most of them in the text.
- **Reviewer:** The authors state: "OA is a degenerative process involving the progressive loss of structural features and functionality of the articular cartilage caused by an imbalance between anabolic and catabolic processes in the cartilage tissue, so that cartilage degradation exceeds reparative processes and OA progresses" - this is not sufficiently precise, since the subchondral bone is also involved in pathogenesis of OA. The authors should also mention this and further on, comment on the effects of weight loss/gain and exercise training on bone mineral density in OA patients.
**Response: **We have provided to modify the text with comment on the involvement of the subchondral bone in pathogenesis of OA, and on the effects of weight loss/gain and exercise training on bone mineral density in OA patients. Thank you for the paper suggestion.
- **Reviewer: "**Cartilage is a slow adapting tissue, indeed it undergoes 75% adaptation in approximately 2 years." - Reference for this statement?
**Response:** We inserted the following reference: Hambly K, Griva K: IKDC or KOOS? Which measures symptoms and disabilities most important to postoperative articular cartilage repair patients? Am J Sports Med. 2008;36(9):1695-704.
- **Reviewer:** The paragraph under title “Second step post-operative rehabilitation in OA” - the whole paragraph is very repetitive, with the same kind of information provided in the table as well. Can be shortened.
**Response: **We have provided for modifying the text and making it shorter.
- **Reviewer:** It would be good to give short overview of the available surgical procedures to treat OA. The authors mention “Arthroscopic procedures, such as chondroplasty or microfracture, may resolve faster than osteochondral autograph transplantation (OATS) or autologous chondrocyte implantation (ACI) that involve larger incisions, requiring a slower exercise rehabilitation program”, but it would be better if they gave a short description of each procedure (not more than a sentence long).
**Response:** We have provided for modifying the text with a short description of each procedure.

The Authors hope that the reviewer will be satisfied with the changes and revisions made. Thank you for your time and for considering our paper.

# Response

**Published:** 2015-12-30

**Authors:** Giuseppe Musumeci

**Affiliations:** University of Catania, Italy

Thank you for your review and for the comments. Comments from reviewers are all valuable and very helpful for revising and improving our paper. We have studied the comments carefully and have made corrections which we hope meet with your approval.

Point by point replies

Reviewer

It is not very clear how the selection of articles was performed. The authors state: "The literature search was conducted from in March 2014 to April 2015 on PubMed, Scopus, Web of Science and Google Scholar using appropriate keywords (osteoarthritis, rehabilitation, exercise and nutrition). Of approximately 160 papers (original articles, systematic and meta-analysis reviews) only 67 have been chosen and considered appropriate for the purpose of the review. The other papers, have not been considered as they resulted outside the scope of the research." However, out of these 67, only 7 are from 2014 and 3 from 2015. The rest range from 1995-2013 (References). The authors should clarify the selection criteria and perhaps include more of the more recent papers.

Response

The authors thank the reviewer for this comment. Perhaps we have not been clear, we meant to say that we have made the literature search in the period March 2014 - December 2015, but the period considered in the selection of the references is much larger (1995 – 2015). Thank you for letting us know about the possible misinterpretation. We have corrected the text to be clearer. Thank you for the papers suggested, we included most of them in the text.

Reviewer

The authors state: "OA is a degenerative process involving the progressive loss of structural features and functionality of the articular cartilage caused by an imbalance between anabolic and catabolic processes in the cartilage tissue, so that cartilage degradation exceeds reparative processes and OA progresses" - this is not sufficiently precise, since the subchondral bone is also involved in pathogenesis of OA. The authors should also mention this and further on, comment on the effects of weight loss/gain and exercise training on bone mineral density in OA patients.

Response

We have provided to modify the text with comment on the involvement of the subchondral bone in pathogenesis of OA, and on the effects of weight loss/gain and exercise training on bone mineral density in OA patients. Thank you for the paper suggestion.

Reviewer

"Cartilage is a slow adapting tissue, indeed it undergoes 75% adaptation in approximately 2 years." - Reference for this statement?

Response

We inserted the following reference: Hambly K, Griva K: IKDC or KOOS? Which measures symptoms and disabilities most important to postoperative articular cartilage repair patients? Am J Sports Med. 2008;36(9):1695-704.

Reviewer

The paragraph under title “Second step post-operative rehabilitation in OA” - the whole paragraph is very repetitive, with the same kind of information provided in the table as well. Can be shortened.

Response

We have provided for modifying the text and making it shorter.

Reviewer

It would be good to give short overview of the available surgical procedures to treat OA. The authors mention “Arthroscopic procedures, such as chondroplasty or microfracture, may resolve faster than osteochondral autograph transplantation (OATS) or autologous chondrocyte implantation (ACI) that involve larger incisions, requiring a slower exercise rehabilitation program”, but it would be better if they gave a short description of each procedure (not more than a sentence long).

Response

We have provided for modifying the text with a short description of each procedure.

The Authors hope that the reviewer will be satisfied with the changes and revisions made.

Thank you for your time and for considering our paper.
